# Prevalence of cervical human papillomavirus infections and cytological abnormalities in asymptomatic women living with HIV in Addis Ababa, Ethiopia

**DOI:** 10.1038/s41598-026-64488-7

**Published:** 2026-08-01

**Authors:** Maria Andersson, Bethelehem Nigussie Zewdie, Chrispinus Mumena, Elin Andersen Thulin, Gashaw Garedew Woldeamanuel, Bengt Hasséus, Abdu Mengesha, Daniel Giglio

**Affiliations:** 1 Department of Infectious Diseases, Institute of Biomedicine, Sahlgrenska Academy, University of Gothenburg, Gothenburg, Sweden; 2 Department of Clinical Microbiology, Sahlgrenska University Hospital, Gothenburg, Sweden; 3 Communicable and Non-Communicable Diseases Research Directorate, Armauer Hansen Research Institute, P.O. Box 1005, Addis Ababa, Ethiopia; 4 Department of Oncology, Institute of Clinical Sciences, Sahlgrenska Academy, University of Gothenburg, Blå Stråket 2, Gothenburg, SE-41345 Sweden; 5 Department of Dental Clinical Sciences, School of Medicine, Copperbelt University, Ndola, Zambia; 6 Department of Biomedical Sciences, School of Medicine, Wolkite University, Wolkite, Ethiopia; 7 Department of Oral Medicine and Pathology, Institute of Odontology, Sahlgrenska Academy, University of Gothenburg, Gothenburg, Sweden; 8 Department of Obstetrics and Gynecology, School of Medicine, College of Health Sciences, Addis Ababa University, Addis Ababa, Ethiopia; 9 Department of Medicine and Oncology, Southern Älvsborg Hospital, Borås, Sweden; 10 Department of Pharmacology, Institute of Neuroscience and Physiology, Sahlgrenska Academy, University of Gothenburg, Gothenburg, Sweden

**Keywords:** Human papillomavirus, HIV, Cervical cancer, Ethiopia, Cancer, Diseases, Health care, Medical research, Microbiology, Risk factors

## Abstract

Cervical cancer from persistent HPV infection is the leading cause of cancer death among Sub-Saharan African women. We here addressed the existing knowledge gap concerning the prevalence of HPV infection and cervical lesions in asymptomatic women living with HIV (WLWH) in Addis Ababa, Ethiopia. Adult WLWH (*N* = 334) were recruited at the HIV clinic of the Tikur Anbessa Specialized Hospital, Addis Ababa. Real-time PCR targeting 12 high-risk (HR) and 2 low-risk HPV types on cervical samples and Pap smear testing were performed. Logistic regression was used in IBM SPSS Statistics to identify factors associated with HPV infection and abnormal cytology. HR-HPV infections occurred in 20.6% of participants and the most prevalent HR-HPV genotypes were HPV35 (16.8%) and HPV16 (14.7%). Abnormal cytology was more common among HR-HPV–positive women than HR-HPV–negative women (36.1% vs. 12.6%, *p* < 0.0001). LSIL and HSIL were detected in 9.9% and 6.6% of HR-HPV–positive women, respectively. Condom use was associated with reduced odds of HPV infection and abnormal cytology. HR-HPV infections dominated by non-HPV16/18 types and abnormal cytology were common among asymptomatic WLWH in Ethiopia underscoring the need for strengthened cervical cancer screening, HPV vaccinations and safe-sex education for this high-risk population.

## Introduction

### Cervical cancer screening in Sub-Saharan Africa

Human papillomavirus (HPV)-induced cervical cancer is the leading cause of female cancer death in Sub-Saharan Africa (SSA) and the second leading cause of cancer-related death among women living in Ethiopia^1^. The World Health Organization (WHO) cervical cancer elimination strategy targets 90% HPV vaccination coverage in girls by age 15 years, 70% screening coverage at least twice in a lifetime, and 90% access to treatment for women with detected disease by 2030^2^. Modelling has suggested that cervical cancer can be eliminated by the end of this century in the majority of low- and middle-income countries^3^. Presently, nationwide cervical cancer screening programs are not enrolled in Ethiopia and cervical cancer screening is offered opportunistically to risk groups^4^. Opportunistic screening is often performed with visual inspection with acetic acid (VIA) and the screen-and-treat approach is presently employed. However, a cervical cancer screening study conducted among 17,586 women in rural Ethiopia showed that only one third of VIA-positive women returned for rescreening one year later, showing the difficulty of follow-up adherence^5^. In this study, women living with HIV (WLWH) had a better follow-up adherence than HIV-negative women. This is in contrast to other studies from Ethiopia showing the positive correlation between HIV status and adherence^6^.

### Prevalence of cervical HPV infections among WLWH in Ethiopia

The prevalence of cervical HPV infections in Ethiopian women varies substantially between studies depending on studied cohorts, symptoms, HIV status and location in the country. There is also a variability in the number of HPV types examined between studies. For example, half of WLWH living in Northwestern Ethiopia were positive for cervical HR-HPVs and close to one fourth had cytological abnormalities^7^. In a study conducted in Southern Ethiopia, the percentage of WLWH with HR-HPV infections was 35.2%^8^. The prevalence of precancerous cervical lesions in WLWH attending the Zewditu Memorial Hospital, Addis Ababa, Ethiopia was 16.6%^9^.

The risk of developing cervical cancer is increased six times in WLWH^10^. We previously reported a cervical HR-HPV prevalence of 38.3% and cytological abnormalities in 24.2% of HR-HPV positive cases among asymptomatic WLWH attending the HIV clinic at the University Teaching Hospital of Kigali, Rwanda^11^. At present only few studies have been conducted in Ethiopia on the prevalence of HPV infections in asymptomatic WLWH. In the present study we assessed the prevalence of HPV and cervical cytological abnormalities in WLWH attending the HIV clinic at the Tikur Anbessa Specialized Hospital in Addis Ababa.

## Methods

### Study setting, period, and population

We included 334 WLWH treated at the HIV clinic, Tikur Anbessa Specialized Hospital, Addis Ababa, Ethiopia, who were recruited to the study after informed consent between March 2023 and February 2024. Women were included if they met the following inclusion criteria: ≥21 years of age, HIV positive status, and voluntarily seeking HPV screening. Women were excluded if they met the following exclusion criteria: prior HPV vaccination, a present or previous diagnosis of cervical lesions/cancer, visible or known vaginal or cervical infections (other than HPV) at the time of inclusion, lack of sexual activity with others, or any condition that might prevent compliance with the study protocol. Women were interviewed face to face by nurses at the HIV clinic about medical history, sexual and obstetric history, prior sexual transmitted infections (STIs) and risk factors to contract HPV infections. The questionnaire was adapted from a questionnaire previously used by the research group in a similar study in Rwanda^11^. The wording of questionnaire items and response options was reviewed and discussed within the research group to ensure contextual appropriateness for the Ethiopian setting. After this, women were examined by nurses at the HIV clinic, trained in the sampling technique by gynecologist AM, and a swab sample was taken from the uterine cervix with the Aptima Multitest Swab (Hologic Inc., Marlborough, MA, USA). Pap smear samples were then collected with a wooden spatula and cyto-brush.

### Real-time PCR

Real-time PCR was conducted by using the QuantStudio™ 6 Flex Real-Time PCR System (Applied Biosystems, Foster City, CA, USA) targeting specific segments of the E6/E7 region for the HR-HPV types HPV16, 18, 31, 33, 35, 39, 45, 51, 52, 56, 58 and 59, and the two low-risk HPV (LR-HPV) types HPV6 and 11, and the housekeeping gene beta-globin as previously described^12,13^. This in-house method is regularly used in patient diagnostics to type HPV at the Sahlgrenska University Hospital, Gothenburg, Sweden, due to its sensitivity, as a complement to commercial assays and is yearly verified through participation in external quality control programs.

### Cytology

Pap smears were stained according to the method of Papanicolaou, and cytology was performed in accordance with the 2014 Bethesda System as negative for squamous intraepithelial lesion or malignancy (NILM), atypical squamous cells of undetermined significance (ASCUS), low-grade squamous intraepithelial lesion (LSIL), atypical squamous cells cannot exclude high-grade squamous intraepithelial lesion (ASC-H), atypical glandular cells (AGC), atypical, favor neoplastic, endocervical adenocarcinoma in situ (AIS), high-grade squamous intraepithelial lesion (HSIL), and squamous cell carcinoma (SCC)^14^. The cytological analysis was conducted by a well-experienced pathologist/cytologist (BNZ), who had knowledge only of participant age and was blinded to HPV status.

### Data analysis and statistics

The sample size was initially calculated to compare the HPV prevalence between HIV-positive and HIV-negative women in Addis Ababa. Based on published estimates, we assumed HPV prevalence of 12.5% among HIV-positive women and 5% among HIV-negative women. Using a two-sided alpha of 0.05 and 90% power, the required sample size was 293 participants per group. After accounting for a 10% dropout rate, the final target sample size was 326 participants per group. However, due to operational and logistical challenges in recruiting HIV-negative women within the study setting and timeframe, the study design was revised to focus exclusively on HIV-positive women. The final sample size of 326 HIV-positive women was therefore retained based on the original feasibility target and available resources. Ultimately, 334 HIV-positive women were recruited, slightly exceeding the target sample size.

Continuous variables are given as mean±standard deviation. For categorical variables, bivariate logistic regression analysis was conducted to identify explanatory variables correlating with the presence of HPV infection or cytological abnormalities, and crude odds ratios (COR) were estimated. Identified explanatory variables (*p* < 0.10) were included in a multivariable logistic regression analysis and adjusted odds ratios were estimated. Multicollinearity among independent variables was assessed using the variance inflation factor (VIF). A VIF value < 5 was considered indicative of acceptable collinearity. Significance was considered a P-value less than 0.05. IBM SPSS Statistics version 30.0.0.0 (IBM Corp., Armonk, NY, USA) and GraphPad Prism 9.1.0 (GraphPad Software, Inc., San Diego, USA) were used to analyze the data.

## Results

### Sociodemographics of the cohort and risk factors associated with HPV contraction

The sociodemographic characteristics of the cohort are presented in Table 1. In brief, most of the women (40.8%) were married and the mean age was 43.9 ± 8.5 years (*N* = 334). The largest proportion of women (41.6%) had partially or fully completed primary school only, while 13.9% had no formal education. Unemployment was most common (39.2%) followed by work in the private sector (20.8%). Alcohol use was reported by 29.7% of participants and only 1.1% were current or former smokers. The mean age at first sexual intercourse was 19.2 ± 4.3 years (range 6–40) and 24.3% of women had had more than two lifetime sexual partners. The reported condom use was only 17.8%. Previous infections with gonorrhea, syphilis and chlamydia were reported in 13.5%, 10.8% and 15.7% of participants, respectively. Previous Pap smear testing had been performed in 27.7% of participants.

**Table 1:** Characteristics of the study participants (*N* = 334).

	Number	%
**Age groups (years)**		
≤ 35	36	11.1
36–45	152	46.8
46–55	116	35.7
> 55	21	6.5
Missing data	9	
**Marital status**		
Married	135	40.8
Male partner, unmarried	17	5.1
Female partner, unmarried	3	0.9
Divorced/separated	72	21.8
Widow	79	23.9
Single	25	7.6
Missing data	3	
**Educational level**		
No formal education	46	13.9
Primary school (incomplete-complete)	138	41.6
Secondary school (incomplete-complete)	98	29.5
University (incomplete-complete)	49	14.8
Other	1	0.3
Missing data	2	
**Occupation**		
Farming	1	0.3
Civil servant	47	14.2
Business	37	11.1
Community health worker	8	2.4
Unemployed	130	39.2
Private sector	69	20.8
Other	40	12.0
Missing data	2	
**Smoking**		
No	324	98.9
Yes, current smoker	1	0.3
Ex-smoker	3	0.9
Missing data	6	
**Alcohol use**		
Never/seldom	230	70.3
Once a month	85	26.0
Once a week	10	3.1
Several times a week	2	0.6
Missing data	7	
**Serious disease**		
No	240	73.2
Yes	88	26.8
Missing data	6	
**If yes**,** specify**		
Cardiovascular disease	30	36.5
Cancer	4	4.9
Rheumatic disease	3	3.7
Other serious disease*	53	64.6
Missing data	6	
**Age at first sexual intercourse (years)**		
≤ 14	33	10.9
15–17	71	23.5
18–21	133	44.0
≥ 22	65	21.5
Missing data	32	
**Number of sex partners**		
0	1	0.3
1–2	248	75.4
≥ 3	80	24.3
Missing data	5	
**Contraceptive method**		
Sterilization	2	0.6
Male partner sterilized	4	1.3
Contraceptive implant/injectable	71	22.2
Contraceptive pill	64	20.0
Male condom	57	17.8
Rhythm method	4	1.3
Other method	12	3.8
Not using	157	49.0
Missing data	14	
**Past gonorrhea infection**		
No	274	84.3
Yes	44	13.5
I don´t know	7	2.2
Missing data	9	
**Past syphilis infection**		
No	285	87.7
Yes	35	10.8
I don´t know	9	2.8
Missing data	9	
**Past chlamydia infection**		
No	260	80.0
Yes	51	15.7
I don´t know	14	4.3
Missing data	9	
**Previous pap smear**		
Yes	91	27.7
No	227	69.0
I don’t know	11	3.3
Missing data	5	
**CD4 count (cells/ml)**		
< 200	88	33.0
200–500	90	33.7
> 500	89	33.3
Missing data	67	
**HIV viral load (copies/ml)**		
< 400	289	97.0
400–5000	7	2.3
> 5000	2	0.7
Missing data	36	

* Including neurological/psychiatric conditions (20.7%), respiratory conditions/infections (12.2%), diabetes (12.2%), renal failure (4.9%) and other conditions/diseases (9.8%).

### Prevalence of HPV types and cytological abnormalities

Single and multiple HR-HPV infections occurred in 13.9% and 6.7% of cases, respectively (Table 2). No LR-HPV infections (HPV6 and HPV11) were detected among the participants. The most prevalent HR-HPV infections (% of all infections) were HPV35 (18.1%), HPV16 (13.8%), HPV58 (10.6%), HPV51 (9.6%) and HPV56 (9.6%). Among HR-HPV infected women, 36.1% had abnormal cytology compared with 12.6% of HR-HPV negative women (*P* < 0.0001; Table 3). LSIL was detected in 9.9% and HSIL in 6.6% of HR-HPV positive women while the corresponding figures in HPV-negative were only 0.4% and 0.4%, respectively. In women with abnormal cytology, 5.9% were positive for HPV16, 8.8% for HPV18 and 85.3% for non HPV16/18 types.

**Table 2:** High-risk and low-risk HPV types in the cohort (*N* = 334).

High-risk HPV status	*N*	%
Negative	250	79.4
One HR-HPV type	44	13.9
Multiple HR-HPV types	21	6.7
Missing data	19	
**High-risk HPV types**		**% of all infections**
HPV16	13	13.8
HPV18	6	6.4
HPV31	7	7.4
HPV33	3	3.2
HPV35	17	18.1
HPV39	7	7.4
HPV45	3	3.2
HPV51	9	9.6
HPV52	7	7.4
HPV56	9	9.6
HPV58	10	10.6
HPV59	3	3.2
**Low-risk HPV types**		
HPV6	0	0
HPV11	0	0

**Table 3:** High-risk HPV and cytology.

Pap smear interpretation	HR-HPV positive *N* (%)	HR-HPV negative *N* (%)	Significance
NILM	39 (63.9)	208 (87.4)	*P* < 0.0001
Abnormal	22 (36.1)	30 (12.6)	
ASCUS	8 (13.1)	19 (8.0)	
ASC-H	4 (6.6)	8 (3.4)	
AGUS	0 (0)	1 (0.4)	
LSIL	6 (9.8)	1 (0.4)	
HSIL	4* (6.6)	1 (0.4)	
Inadequate	2	5	
Missing data	2	7	

*One patient had cytological findings consistent with either HSIL or SCC. Chi-square test was used between NILM and abnormal cytology (ASCUS, ASC-H, AGUS, HSIL or LSIL).

### Factors associated with HPV infections and cytological abnormalities

The associations between covariates and development of cervical HPV infection(s) and abnormal cytology are displayed in Table 4. Occupation had no significant association with HPV infections or abnormal cytology, however, employment in private or informal economic activity compared with public or community services was associated with reduced odds of HPV infection (COR 0.46, 95% CI: 0.22–0.96, *P* = 0.038) and abnormal cytology (COR 0.43, 95% CI: 0.18–1.02, *P* = 0.056). Non-use of contraceptive methods compared with condom use tended to increase the odds of HPV infection (COR 2.37, 95% CI: 0.99–5.68, *P* = 0.053). Use of contraceptive methods other than condoms as well as non-use of contraception were associated with an increased odds of abnormal cytology (COR 5.56, 95% CI: 1.23–25.06, *P* = 0.025 and COR 7.54, 95% CI: 1.74–32.59, *P* = 0.0068, respectively). No other variables were significantly associated with HPV infection or abnormal cytology. Neither were any variables significant in multivariable analysis, however, opting not using condoms tended to be associated with the development of abnormal cytology (*p* = 0.071; Table 4).

**Table 4:** Associations between covariates and presence of cervical HPV infection(s) and abnormal cytology.

	Present cervical HPV infection(s)			Present abnormal cytology		
	HPV+ *N* (%)	Crude OR (95% CI)	*P*-value	Abnormal cytology *N* (%)	Crude OR (95% CI)	*P*-value
**Age groups**			0.313			0.874
≤ 35	4 (11.8)	Ref.		5 (14.3)	Ref.	
36–45	27 (18.6)	1.72 (0.56–5.28)	0.346	25 (17.5)	1.27 (0.45–3.60)	0.651
46–55	24 (22.2)	2.14 (0.69–6.68)	0.189	18 (16.5)	1.19 (0.41–3.47)	0.754
> 55	6 (31.6)	3.46 (0.83–14.36)	0.087	2 (10.0)	0.67 (0.12–3.80)	0.648
Missing data	3 (37.5)	4.50 (0.77–26.45)	0.096	2 (25.0)	2.00 (0.31–12.84)	0.465
**Marital status**			0.648			0.702
Married/partner	26 (17.4)	Ref.		22 (14.9)	Ref.	
Divorced/separated	14 (21.2)	1.27 (0.62–2.63)	0.514	13 (20.6)	1.49 (0.70–3.18)	0.304
Widow	17 (23.3)	1.44 (0.72–2.86)	0.303	12 (15.6)	1.06 (0.49–2.27)	0.886
Single	6 (26.1)	1.67 (0.60–4.64)	0.326	5 (20.8)	1.51 (0.51–4.46)	0.458
**Educational level**			0.578			
No formal education-primary school	38 (22.1)	1.29 (0.74–2.27)	0.371	28 (16.4)	0.96 (0.53–1.73)	0.879
> Primary school	25 (18.0)	Ref.		24 (17.0)	Ref.	
Missing data/other	1 (33.3)	2.28 (0.20–26.14)	0.508			
**Occupation**			0.155			0.050
Public & Community Services^A^	16 (30.2)	Ref.		11 (20.8)	Ref.	
Private & Informal Economic Activity^B^	23 (16.5)	0.46 (0.22–0.96)	0.038	14 (10.1)	0.43 (0.18–1.02)	0.056
Unemployed	24 (20.0)	0.58 (0.28–1.21)	0.145	26 (21.3)	1.03 (0.47–2.28)	0.934
Missing data	1 (50.0)	2.31 (0.14–39.31)	0.562	1 (50.0)	3.82 (0.22–66.02)	0.357
**Alcohol use**			0.294			0.940
Never/seldom	40 (18.9)	Ref.		35 (16.1)	Ref.	
Yes	21 (22.1)	1.22 (0.67–2.21)	0.511	16 (17.6)	1.11 (0.58–2.12)	0.754
Missing data	3 (42.9)	3.22 (0.69–14.98)	0.135	1 (14.3)	0.87 (0.10–7.42)	0.896
**Serious disease**			0.974			
No	46 (20.4)	Ref.		40 (17.6)	Ref.	
Yes	17 (20.5)	1.00 (0.54–1.87)	0.994	12 (14.6)	0.80 (0.40–1.62)	0.536
Missing data	1 (16.7)	0.78 (0.089–6.83)	0.821			
**Age at first sexual intercourse**			0.653			0.157
**<** 18	21 (21.4)	1.03 (0.57–1.88)	0.910	13 (13.4)	0.64 (0.32–1.26)	0.196
≥ 18	39 (20.9)	Ref.		37 (19.6)	Ref.	
Missing data	4 (13.8)	0.61 (0.20–1.85)	0.380	2 (6.9)	0.30 (0.07–1.34)	0.115
**Number of sex partners**			0.570			
0–2	47 (20.2)	Ref.		41 (17.3)	Ref.	
≥ 3	15 (19.7)	0.97 (0.51–1.86)	0.934	11 (15.1)	0.85 (0.41–1.75)	0.656
Missing data	2 (40.0)	2.64 (0.43–16.24)	0.296			
**Contraceptive method**			0.161			0.023
Male condom	7 (12.7)	Ref.		2 (3.6)	Ref.	
Other method(s)	17 (17.0)	1.40 (0.54–3.63)	0.483	17 (17.3)	5.56 (1.23–25.06)	0.025
Not using	38 (25.7)	2.37 (0.99–5.68)	0.053	33 (22.1)	7.54 (1.74–32.59)	0.0068
Missing data	2 (18.2)	1.52 (0.27–8.55)	0.632			
**Past gonorrhea infection**			0.723			
No	54 (21.1)	Ref.		49 (18.8)	Ref.	
Yes	6 (14.3)	0.62 (0.25–1.56)	0.311	3 (7.9)	0.37 (0.11–1.26)	0.111
I don´t know	2 (28.6)	1.50 (0.28–7.93)	0.636			
Missing data	2 (22.2)	1.07 (0.22–5.29)	0.935			
**Past syphilis infection**			0.634			
No	55 (20.8)	Ref.		47 (17.5)	Ref.	
Yes	5 (16.1)	0.73 (0.27–2.00)	0.546	5 (17.2)	0.98 (0.36–2.70)	0.968
I don´t know	3 (33.3)	1.91 (0.46–7.88)	0.371			
Missing data	1 (11.1)	0.48 (0.058–3.90)	0.490			
**Past chlamydia infection**			0.983			
No	50 (20.7)	Ref.		43 (17.6)	Ref.	
Yes	9 (18.4)	0.86 (0.39–1.90)	0.716	9 (18.9)	1.08 (0.49–2.40)	0.842
I don´t know	3 (21.4)	1.05 (0.28–3.90)	0.945			
Missing data	2 (22.2)	1.10 (0.22–5.44)	0.910			
**Previous pap smear**			0.740			0.987
Yes	21 (24.4)	Ref.		14 (15.9)	Ref.	
No	40 (18.7)	0.71 (0.39–1.30)	0.266	37 (17.5)	1.12 (0.57–2.19)	0.746
I don´t know	2 (22.2)	0.88 (0.17–4.59)	0.884	1 (20.0)	1.32 (0.14–12.72)	0.809
Missing data	1 (20.0)	0.77 (0.082–7.31)	0.823			
**CD4 count (cells/ml)**			0.176			0.730
< 200	18 (21.7)	0.78 (0.38–1.59)	0.496	13 (16.3)	1.42 (0.64–3.15)	0.383
200–500	11 (12.6)	0.41 (0.18–0.91)	0.028	17 (20.2)	1.09 (0.47–2.52)	0.841
> 500	22 (26.2)	Ref.		13 (15.1)	Ref.	
Missing data	13 (21.7)	0.78 (0.36–1.71)	0.533	9 (14.1)	0.90 (0.36–2.26)	0.827
				**Abnormal cytology** **N (%)**	**Adjusted OR** **(95% CI)**	**P-value/VIF**
**Occupation**						0.106/1.037
Public & Community Services^A^				11 (20.8)	Ref.	
Private & Informal Economic Activity^B^				14 (10.1)	0.44 (0.18–1.05)	0.064
Unemployed				26 (21.3)	1.03 (0.46–2.31)	0.951
Missing data				1 (50.0)	Undetermined	
**Contraceptive method**						0.071/1.037
Male condom				2 (3.6)	Ref.	
Other method(s)				17 (17.3)	5.93 (1.31–26.93)	0.021
Not using				33 (22.1)	7.28 (1.67–31.68)	0.0082

Percentages represent the proportion of women with HPV infections and abnormal cytology, respectively, within each category. ^A^Civil servant and community health worker; ^B^Business, private sector and other. CI: confidence interval; VIF: variance inflation factor.

## Discussion

We show a substantial prevalence of HR-HPV infections and cytological abnormalities in WLWH in Addis Ababa, Ethiopia. HPV35 was the most common HPV type in our cohort and non-HPV16/18 dominated among HPV types. There was a low uptake of cervical screening, and the use of condoms was low, highlighting important gaps in prevention.

### Prevalence of HR-HPV among WLWH in Ethiopia

The prevalence of HR-HPV infection in our cohort (20.6%) was lower than that reported among WLWH recruited from health facilities in Addis Ababa (28.7%), from hospitals in Addis Ababa (23.9–29.4%) and Northeast Ethiopia (28.8%)^15–17^. It was also significantly lower than the 38.3% prevalence of HR-HPV observed in a parallel study of WLWH attending an HIV clinic in Kigali, Rwanda^11^. Notably, that study was conducted using the same protocol as the present investigation, suggesting that population differences rather than methodological variation may explain the observed discrepancy.

### HPV genotype distribution and implications for vaccination

In line with previous studies in WLWH in Ethiopia showing prevalence of non-HPV16/18 of 48–73%^7,15,16^, non-HPV16/18 types dominated in our cohort (79%). As demonstrated for other African countries^18^, HPV35 was prevalent in our cohort compared to other regions in the world. This HPV type was shown to be particularly common in HSIL and cervical cancer in South African women^19^. In our study, HPV58, HPV51 and HPV56 were also among the top prevalent HPV types. Similarly, HPV58 and HPV51 were also in the top of cervical HPV infections in WLWH in Rwanda^11^. The current use of the quadrivalent Gardasil HPV vaccine targeting solely HPV16 and HPV18 among HR-HPV types, may therefore provide incomplete coverage even if this vaccine may give partial cross-protection against HPV31 and HPV45^20,21^. Implementation of HPV vaccinations giving a fuller HPV coverage, such as Gardasil-9, would be beneficial to further reduce the number of cervical cancer cases in Ethiopia in the future. Nevertheless, HPV35 is not covered by the Gardasil-9 vaccine. We could not detect any women with HPV6 or HPV11 in our cohort in contrast to our study in WLWH in Rwanda where these types were detected at a low frequency^11^. As shown previously, the prevalence of cervical infections by LR-HPV types is low in Ethiopian women compared to HR-HPV types^22^.

### Sexual, reproductive, and behavioral risk factors

Only 27.7% of women had previously been screened with Pap smear tests. This uptake is higher than the 18.2% uptake observed in a meta-analysis in WLWH in Ethiopia^23^. This is, however, half the uptake of our parallel study conducted in WLWH in Rwanda^11^. To note, we did not presently assess the prevalence of other screening methods such as VIA. Non-use of condoms was associated with an increased risk of cervical HPV infection and cytological abnormalities as previously demonstrated among Ethiopian WLWH^16^.

Our study suggests that employment status may be associated with the risk of HPV acquisition and the development of cervical cytological abnormalities as previously been shown^24^. Employment may act as a proxy for socioeconomic status, access to healthcare services, or behavioral factors related to HPV exposure.

### HPV screening

Cervical cancer screening is of particular importance for WLWH. In our cohort, we observed that HPV-positive participants had a close to three times higher risk of cytological abnormalities compared to HPV-negative participants. Moreover, participants with cervical HR-HPV infections had a 16.4% risk of LSIL or worse compared to 0.8% for those who were HR-HPV negative. This suggests that HPV screening could be used also in the present settings to identify women at risk of cervical cancer. While HPV screening is more efficient as a primary cervical screening method, its use is constrained in low-resource settings due to limited laboratory capacities and the need of return visits for delivery of result and treatment, in contrast to the screen-and-treat approach with for example VIA^25^.

### Strength and limitations

The strength of our study is that it is one of few studies on the prevalence of cervical HPV infections and cytological changes in asymptomatic WLWH in Ethiopia. Among limitations of the study is that our study was a single-center study. Furthermore, cytology was not confirmed by histology. Previous Pap smear tests were only assessed and not whether women had undergone a previous VIA screening, which could have led to an underestimation of the proportion of women who had undergone previous cervical screening. Moreover, many of the known risk factors for contraction of HPV and cytological abnormalities were not significant, which most likely was due to power issues. Finally, a substantial proportion of participants had missing data, particularly for HIV viral load and CD4 count, which were missing in 20.4% and 10.8% of participants, respectively.

In conclusion, cervical high-risk HPV infections and cytological abnormalities were common among WLWH in Ethiopia. Most detected high-risk HPV types were non-HPV16/18 genotypes, which are not covered by the quadrivalent Gardasil vaccine currently used in the country. These findings underscore the importance of consistent condom use to reduce the transmission of STIs, including HPV, and highlight the urgent need to strengthen cervical cancer vaccination and screening programs in Ethiopia and public awareness of protection against STIs.

## Data Availability

Additional data can be requested by sending a request to the Institute of Clinical Sciences at the Sahlgrenska Academy, University of Gothenburg, Medicinaregatan 3 A SE-413 90 Göteborg, Sweden, email: klinvet@gu.se.
